# Milloux Inequality of *E*-Valued Meromorphic Function

**DOI:** 10.1155/2014/861573

**Published:** 2014-01-30

**Authors:** Zhaojun Wu, Zuxing Xuan

**Affiliations:** ^1^School of Mathematics and Statistics, Hubei University of Science and Technology, Xianning 437100, China; ^2^Beijing Key Laboratory of Information Service Engineering, Department of General Education, Beijing Union University, No. 97 Bei Si Huan Dong Road, Chaoyang District, Beijing 100101, China

## Abstract

The main purpose of this paper is to establish the Milloux inequality of *E*-valued meromorphic function from the complex plane *ℂ* to an infinite dimensional complex Banach space *E* with a Schauder basis. As an application, we study the Borel exceptional values of an *E*-valued meromorphic function and those of its derivatives; results are obtained to extend some related results for meromorphic scalar-valued function of Singh, Gopalakrishna, and Bhoosnurmath.

## 1. Introduction

In the 1970s, the Nevanlinna theory of meromorphic function is extended to the vector-valued meromorphic function from the complex plane *ℂ* to a finite dimensional space *ℂ* ^*n*^ (see Ziegler [1]). After that, some works related to vector-valued meromorphic function in finite dimensional spaces were done by [2–5]. In 2006, C. G. Hu and Q. J. Hu [6] established Nevanlinna's first and second fundamental theorems for an *E*-valued meromorphic function from the disk *ℂ*_*r*_ = {|*z* | <*r*}, 0 < *r* ≤ +*∞*, to infinite-dimensional Banach spaces *E* with a Schauder basis. Xuan and Wu [7] established Nevanlinna's first and second fundamental theorems for an *E*-valued meromorphic function from a generic domain *D*⊆*ℂ* to *E* and generalized Chuang's inequality. Motivated by [6, 7], Bhoosnurmath and Pujari [8] studied the *E*-valued Borel exceptional values of meromorphic functions, Wu and Xuan [9, 10] studied the characteristic functions, exceptional values, and deficiency of *E*-valued meromorphic function, and Hu [11] surveyed the advancements of the Nevanlinna theory of *E*-valued meromorphic functions and studied its related Paley problems. In this paper, we will generalize Milloux's inequality (see [12] or [13]) to *E*-valued meromorphic function.

## 2. The Nevanlinna Theory in Banach Spaces

In this section, we introduce some fundamental definitions and notations of *E*-valued meromorphic function which was introduced by C. G. Hu and Q. J. Hu [6]. See also [7–10].

Let (*E*, ||•||) be an infinite dimension complex Banach space with Schauder basis {*e*_*j*_} and the norm ||•||. Thus an *E*-valued meromorphic function *f*(*z*) defined in *ℂ*_*r*_, 0 < *r* ≤ +*∞*, can be written as

(1) $$\begin{array}{c} f\left(z\right)=\left(f_{1}\left(z\right),f_{2}\left(z\right),\ldots ,f_{k}\left(z\right),\ldots \right)\in E, \end{array}$$

where *f*_1_(*z*), *f*_2_(*z*),…, *f*_*k*_(*z*),… are the component functions of *f*(*z*). Let *E*_*n*_ be an *n*-dimensional projective space of *E* with a basis {*e*_*j*_}_1_^*n*^. The projective operator *P*_*n*_ : *E* → *E*_*n*_ is a realization of *E*_*n*_ associated with the basis.

The elements of *E* are called vectors and are usually denoted by letters from the alphabet: *a*, *b*, *c*,…. The symbol 0 denotes the zero vector of *E*. We denote vector infinity, complex number infinity, and the norm infinity by $\hat{\infty }$, *∞*, and +*∞*, respectively. A vector-valued mapping is called holomorphic (meromorphic) if all component functions of *f*(*z*) are holomorphic (some of component functions of *f*(*z*) are meromorphic). The *j*th derivative of *f*(*z*) is defined by

(2) $$\begin{array}{c} f^{\left(j\right)}\left(z\right)=\left(f_{1}^{\left(j\right)}\left(z\right),f_{2}^{\left(j\right)}\left(z\right),\ldots ,f_{k}^{\left(j\right)}\left(z\right),\ldots \right), \end{array}$$

where *j* = 1,2,…. A point *z*_0_ ∈ *ℂ*_*r*_ is called a pole (or $\hat{\infty }$ point) of *f*(*z*) if *z*_0_ is a pole (or *∞* point) of at least one of the component functions of *f*(*z*). A point *z*_0_ ∈ *ℂ*_*r*_ is called a zero of *f*(*z*) if *z*_0_ is a common zero of all the component functions of *f*(*z*). A point *z*_0_ ∈ *ℂ*_*r*_ is called a pole or an $\hat{\infty }$-point of *f*(*z*) of multiplicity *q* ∈ *ℕ* ^+^ which means that in such a point *z*_0_ at least one of the meromorphic component functions of *f*(*z*) has a pole of this multiplicity in the ordinary sense of function theory. A point *z*_0_ ∈ *ℂ*_*r*_ is called a zero of *f*(*z*) of multiplicity *q* ∈ *ℕ* ^+^ which means that in such a point *z*_0_ all component functions of *f*(*z*) vanish, each with at least this multiplicity.

An *E*-valued meromorphic function *f*(*z*) in *ℂ* is said to be of compact projection, if for any given *ɛ* > 0, ||*P*_*n*_(*f*(*z*)) − *f*(*z*)|| < *ɛ* as sufficiently large *n* in any fixed compact subset *D* ⊂ *ℂ*.

Let *n*(*r*, *f*) or $n(r,\hat{\infty })$ denote the number of poles of *f*(*z*) in |*z* | ≤*r* and *n*(*r*, *a*, *f*) denote the number of *a*-points of *f*(*z*) in |*z* | ≤*r*, counting with multiplicities. Define the volume function associated with *E*-valued meromorphic function *f*(*z*) by

(3) V(r,∞^,f)=V(r,f)=12π∫Crlog⁡|rξ|Δlog⁡||f(ξ)||dx∧dy,ξ=x+iy;V(r,a,f)=12π∫Crlog⁡|rξ|Δlog⁡||f(ξ)−a||dx∧dy,ξ=x+iy,

and the counting function of finite or infinite *a*-points by

(4) $$\begin{array}{c} N\left(r,f\right)=n\left(0,f\right)\log r+\overset{r}{\underset{0}{\int }}\frac{n\left(t,f\right)-n\left(0,f\right)}{t}dt,\\ N\left(r,\hat{\infty }\right)=n\left(0,\hat{\infty }\right)\log r+\overset{r}{\underset{0}{\int }}\frac{n\left(t,\hat{\infty }\right)-n\left(0,\hat{\infty }\right)}{t}dt,\\ N\left(r,a,f\right)=n\left(0,a,f\right)\log r+\overset{r}{\underset{0}{\int }}\frac{n\left(t,a,f\right)-n\left(0,a,f\right)}{t}dt, \end{array}$$

respectively. Next, we define

(5) $$\begin{array}{c} m\left(r,f\right)=m\left(r,\hat{\infty },f\right)=\frac{1}{2\pi }\overset{2\pi }{\underset{0}{\int }}\log^{+}||f\left(re^{i\theta }\right)||d\theta ;\\ m\left(r,a,f\right)=\frac{1}{2\pi }\overset{2\pi }{\underset{0}{\int }}\log^{+}\frac{1}{||f\left(re^{i\theta }\right)-a||}d\theta ;\\ T\left(r,f\right)=m\left(r,f\right)+N\left(r,f\right). \end{array}$$

Let $\bar{n}(r,f)$ or $\bar{n}(r,\hat{\infty })$ denote the number of poles of *f*(*z*) in |*z* | ≤*r* and $\bar{n}(r,a,f)$ denote the number of *a*-points of *f*(*z*) in |*z* | ≤*r*, ignoring multiplicities. Similarly, we can define the counting functions $\;\bar{N}(r,f)$, $\bar{N}(r,\hat{\infty })$, and $\bar{N}(r,a,f)$ of $\bar{n}(r,f)$, $\bar{n}(r,\hat{\infty })$, and $\bar{n}(r,a,f)$.

Let *f*(*z*)  (*z* ∈ *ℂ*_*r*_) be an *E*-valued meromorphic function and *a* ∈ *E*; if *k* is a positive integer, let ${\bar{n}}_{k}(r,f)$ or ${\bar{n}}_{k}(r,\hat{\infty })$ denote the number of distinct poles of *f*(*z*) of order ≤*k* in |*z* | ≤*r* and ${\bar{n}}_{k}(r,a,f)$ denote the number of distinct *a*-points of *f*(*z*) of order ≤*k* in |*z* | ≤*r*. Similarly, we can define the counting functions $\;{\bar{N}}_{k}(r,f)$, ${\bar{N}}_{k}(r,\hat{\infty })$, and ${\bar{N}}_{k}(r,a,f)$ of ${\bar{n}}_{k}(r,f)$, $\;{\bar{n}}_{k}(r,\hat{\infty })$, and ${\bar{n}}_{k}(r,a,f)$.

If *f*(*z*) is an *E*-valued meromorphic function in the whole complex plane, then the order and the lower order of *f*(*z*) are defined by

(6) $$\begin{array}{c} \lambda \left(f\right)=\underset{r\to \infty }{\mathrm{limsup}}\frac{\log^{+}T\left(r,f\right)}{\log r};\\ \mu \left(f\right)=\underset{r\to \infty }{\mathrm{liminf}}\frac{\log^{+}T\left(r,f\right)}{\log r}. \end{array}$$

We call the *E*-valued meromorphic function *f* admissible if

(7) $$\begin{array}{c} \underset{r\to +\infty }{\mathrm{limsup}}\frac{T\left(r,f\right)}{\log r}=+\infty . \end{array}$$

**Definition 1**

Let *f*(*z*) be an admissible *E*-valued meromorphic function in *ℂ*. One denotes by *S*(*r*, *f*) any quantity such that

(8) $$\begin{array}{c} S\left(r,f\right)=O\left(\log T\left(r,f\right)+\log r\right),\;r⟶+\infty , \end{array}$$

without restriction if *f*(*z*) is of finite order and otherwise except possibly for a set of values of *r* of finite linear measure.

In 2006, C. G. Hu and Q. J. Hu [6] proved the following theorems.

**Theorem 2 A (the *E*-valued Nevanlinna's first fundamental theorem)**

Let *f*(*z*) be a nonconstant *E*-valued meromorphic function in *ℂ*_*R*_ = {|*z* | <*R*}, 0 < *R* ≤ +*∞*. Then, for 0 < *r* < *R*, *a* ∈ *E*, and *f*(*z*)≢*a*,

(9) T(r,f)=V(r,a)+N(r,a)+m(r,a)+log⁡+||cq(a)||+ɛ(r,a).

Here *ɛ*(*r*, *a*) is a function such that

(10) $$\begin{array}{c} \left|ɛ\left(r,a\right)\right|\leq \log^{+}||a||+\log2,\;\;ɛ\left(r,0\right)\equiv 0, \end{array}$$

and *c*_*q*_(*a*) ∈ *E* is the coefficient of the first term in the Laurent series at the point *a*.

**Theorem 2 B (the *E*-valued Nevanlinna's second fundamental theorem)**

Let *f*(*z*) be an admissible *E*-valued meromorphic function of compact projection in *ℂ*_*R*_ = {|*z* | <*R*}, 0 < *R* ≤ +*∞*, and *a* ^[*k*]^ ∈ *E* (*k* = 1,2,…, *q*) be *q* ≥ 3 distinct points. Then, for 0 < *r* < *R*,

(11) $$\begin{array}{c} \overset{q}{\underset{k=1}{\sum }}m\left(r,a^{\left[k\right]},f\right)\leq 2T\left(r,f\right)-N_{1}\left(r\right)+S\left(r,f\right), \end{array}$$

where *N*_1_(*r*) = 2*N*(*r*, *f*) − *N*(*r*, *f*′) + *N*(*r*, 0, *f*′).

## 3. Milloux Inequality of *E*-Valued Meromorphic Function

In this section, we will establish the Milloux inequality of *E*-valued meromorphic function and prove the following theorems.

**Theorem 2 (Milloux inequality)**

Suppose that *f*(*z*) is an admissible *E*-valued meromorphic function of compact projection in *ℂ*_*R*_ = {|*z* | <*R*}, 0 < *R* ≤ +*∞*. Let *a*, *b* ∈ *E* be distinct points and *b* ≠ 0. Then, for 0 < *r* < *R*,

(12) T(r,f)≤N¯(r,f)+(k+1){N¯(r,a,f)+V(r,a,f)}+{N¯(r,b,f(k))+V(r,b,f(k))}+S(r,f).

In order to prove Theorem 2, we will prove the following general form of Milloux inequality of *E*-valued meromorphic function when the multiple values are considered.

**Theorem 3 (general form of Milloux inequality)**

Suppose that *f*(*z*) is an admissible *E*-valued meromorphic function of compact projection in *ℂ*_*R*_ = {|*z* | <*R*}, 0 < *R* ≤ +*∞*. Let *a* ^[*i*]^, *b* ^[*j*]^ ∈ *E* (*i* = 1,2,…, *p*; *j* = 1,2,…, *q*) be distinct points such that *b* ^[*j*]^ ≠ 0  (*j* = 1,2,…, *q*) and let *m*_*i*_, *n*_*j*_  (*i* = 1,2,…, *p*; *j* = 1,2,…, *q*), and *l* be any positive integers. Then

(13) {pq−(∑i=1pkq+1mi+1      +∑j=1q1nj+1+1l+1(1+k∑j=1q1nj+1))}T(r,f) ≤ll+1(1+k∑j=1q1nj+1)N¯l(r,f)  +(kq+1)∑i=1p{N¯mi(r,a[i],f)+V(r,a[i],f)}  +∑j=1q{N¯nj(r,b[j],f(k))+V(r,b[j],f(k))}+S(r,f).

By letting *p* = *q* = 1 and *l*, *m*_*i*_, *n*_*j*_ tend to infinity in (13), we can get Theorem 2. In order to prove Theorem 3, we need the following lemma.

**Lemma 4 (see [10])**

Let *f*(*z*) be of compact projection in *ℂ*; then, for a positive integer *k*, one has

(14) $$\begin{array}{c} \frac{1}{2\pi }\overset{2\pi }{\underset{0}{\int }}\log^{+}\frac{||f^{\left(k\right)}\left(re^{i\theta }\right)||}{||f\left(re^{i\theta }\right)||}d\theta =S\left(r,f\right). \end{array}$$

We are now in the position to prove Theorem 3.

**Proof**

We set

(15) $$\begin{array}{c} F\left(z\right)=\overset{p}{\underset{i=1}{\sum }}\frac{1}{||f\left(z\right)-a^{\left[i\right]}||}; \end{array}$$

then

(16) 12π∫02πlog⁡+F(reiθ)dθ ≤m(r,0,f(k))+12π∫02πlog⁡+{F(reiθ)||f(k)(reiθ)||}dθ.

By [6], we have

(17) $$\begin{array}{c} \frac{1}{2\pi }\overset{2\pi }{\underset{0}{\int }}\log^{+}F\left(re^{i\theta }\right)d\theta \geq \overset{p}{\underset{i=1}{\sum }}m\left(r,a^{\left[i\right]}\right)-\log^{+}\frac{2q}{\delta }. \end{array}$$

From (16) and (17), we can get

(18) ∑i=1pm(r,a[i],f) ≤m(r,0,f(k))+12π∫02πlog⁡+{F(reiθ)||f(k)(reiθ)||}dθ  +log⁡+2qδ.

Hence, we can get from the above inequality and Lemma 4 that

(19) $$\begin{array}{c} \overset{p}{\underset{i=1}{\sum }}m\left(r,a^{\left[i\right]},f\right)\leq m\left(r,0,f^{\left(k\right)}\right)+S\left(r,f\right). \end{array}$$

It follows from Theorem A that

(20) T(r,f(k))=m(r,0,f(k))+N(r,0,f(k))+V(r,0,f(k))+O(1).

Thus from (19) and (20) we deduce

(21) ∑i=1pm(r,a[i],f)≤T(r,f(k))−N(r,0,f(k))−V(r,0,f(k))+S(r,f).

By Theorem A, we have

(22) pT(r,f)≤T(r,f(k))+∑i=1p[N(r,a[i],f)+V(r,a[i],f)]−N(r,0,f(k))−V(r,0,f(k))+S(r,f).

Now it follows from Theorems A and B and Lemma 4 that

(23) qT(r,f(k)) ≤∑j=1q{N(r,b[j],f(k))+V(r,b[j],f(k))}  +N(r,0,f(k))+V(r,0,f(k))+N(r,f(k))  −(N(r,0,f(k+1))+2N(r,f(k))−N(r,f(k+1)))  +S(r,f(k)) =∑j=1q{N(r,b[j],f(k))+V(r,b[j],f(k))}  +N(r,0,f(k))+V(r,0,f(k))+N(r,f(k+1))  −N(r,f(k))+N(r,0,f(k+1))+S(r,f) ≤∑j=1q{N(r,b[j],f(k))+V(r,b[j],f(k))}  +N(r,0,f(k))+V(r,0,f(k))+N¯(r,f)  −N(r,0,f(k+1))+S(r,f).

It follows from (22) and (23) that

(24) pqT(r,f)≤N¯(r,f)+(q−1){∑i=1pN(r,a[i],f)−N(r,0,f(k))} +{∑i=1pN(r,a[i],f)    +∑j=1qN(r,b[j],f(k))−N(r,0,f(k+1))} +q∑i=1pV(r,a[i],f)+∑j=1qV(r,b[j],f(k))+S(r,f).

A zero of *f* − *a* of order *j* > *k* is a zero of *f* ^(*k*+1)^ of order *j* − (*k* + 1) and a zero of *f* ^(*k*)^ − *b* of order *m* is a zero of *f* ^(*k*+1)^ of order *m* − 1. Moreover, zeros of *f* − *a* of order >*k* are zeros of *f* ^(*k*)^ and so are not zeros of *f* ^(*k*)^ − *b* since *b* ≠ 0. Hence

(25) ∑i=1pN(r,a[i],f)+∑j=1qN(r,b[j],f(k))−N(r,0,f(k+1))  ≤∑i=1pNk+1(r,a[i],f)+∑j=1qN¯(r,b[j],f(k)),∑i=1pN(r,a[i],f)−N(r,0,f(k))≤∑i=1pNk(r,a[i],f).

Substituting (25) to (24), we obtain

(26) pqT(r,f)≤N¯(r,f)+(q−1)∑i=1pNk(r,a[i],f)+∑i=1pNk+1(r,a[i],f)+∑j=1qN¯(r,b[j],f(k))+q∑i=1pV(r,a[i],f)+∑j=1qV(r,b[j],f(k))+S(r,f),

since

(27) Nk(r,a[i],f)  ≤kN¯(r,a[i],f)  ≤kmi+1{miN¯mi(r,a[i],f)+N(r,a[i],f)}  ≤kmi+1{miN¯mi(r,a[i],f)+T(r,f)}+O(1),

(28) Nk+1(r,a[i],f)  ≤(k+1)N¯(r,a[i],f)  ≤k+1mi+1{miN¯mi(r,a[i],f)+N(r,a[i],f)}  ≤k+1mi+1{miN¯mi(r,a[i],f)+T(r,f)}+O(1).

Similarly, we can get

(29) N¯(r,b[j],f(k))  ≤1nj+1{njN¯nj(r,b[j],f(k))+T(r,f(k))}+O(1),N¯(r,f)≤1l+1{lN¯l(r,f)+T(r,f)}.

By Lemma 4, we can get

(30) T(r,f(k))=m(r,f(k))+N(r,f(k))≤m(r,f)+N(r,f(k)) +12π∫02πlog⁡+||f(k)(reiθ)||||f(reiθ)||dθ≤m(r,f)+N(r,f)+kN¯(r,f)+S(r,f)≤T(r,f)+kN¯(r,f)+S(r,f).

Substituting (27)–(30) into (26), we obtain

(31) pqT(r,f) ≤N¯(r,f)+(q−1)  ×∑i=1pkmi+1{miN¯mi(r,a[i],f)+T(r,f)}  +∑i=1pk+1mi+1{miN¯mi(r,a[i],f)+T(r,f)}  +∑j=1q1nj+1{njN¯nj(r,b[j],f(k))+T(r,f(k))}  +q∑i=1pV(r,a[i],f)+∑j=1qV(r,b[j],f(k))+S(r,f) ≤(1+∑j=1qknj+1)N¯(r,f)+(q−1)  ×∑i=1pkmimi+1N¯mi(r,a[i],f)  +∑i=1pk+1mi+1miN¯mi(r,a[i],f)  +∑j=1qnjnj+1N¯nj(r,b[j],f(k))  +(q−1)∑i=1pkmi+1T(r,f)  +∑i=1pk+1mi+1T(r,f)+∑j=1q1nj+1njT(r,f)  +q∑i=1pV(r,a[i],f)+∑j=1qV(r,b[j],f(k))+S(r,f) ≤(1+∑j=1qknj+1)ll+1N¯l(r,f)+(kq+1)  ×∑i=1pmimi+1N¯mi(r,a[i],f)  +∑j=1qnjnj+1N¯nj(r,b[j],f(k))  +q∑i=1pV(r,a[i],f)+∑j=1qV(r,b[j],f(k))  +(∑i=1pkq+1mi+1+∑j=1q1nj+1+1l+1(1+k∑j=1q1nj+1))  ×T(r,f)+S(r,f).

Since *m*_*i*_, *n*_*j*_, *k*, and *q* are positive integers, it follows from (31) that

(32) pqT(r,f)≤(∑i=1pkq+1mi+1+∑j=1q1nj+1+1l+1(1+k∑j=1q1nj+1)) ×T(r,f) +(kq+1)∑i=1p[N¯mi(r,a[i],f)+V(r,a[i],f)] +∑j=1q[N¯nj(r,b[j],f(k))+V(r,b[j],f(k))] +(1+∑j=1qknj+1)ll+1N¯l(r,f)+S(r,f).

Hence, (13) follows from (32).

## 4. *E*-Valued Borel Exceptional Values of Meromorphic Function and Its Derivatives

Most recently, Bhoosnurmath and Pujari [8] studied the *E*-valued Borel exceptional values of meromorphic functions and gave the following definition.

**Definition 5**

Let *f*(*z*)  (*z* ∈ *ℂ*) be an *E*-valued meromorphic function and $a\in E\cup \{\hat{\infty }\}$ *k* is a positive integer. One defines

(33) $$\begin{array}{c} {\bar{\rho }}_{k}\left(a,f\right)=\underset{r\to \infty }{\mathrm{limsup}}\frac{\log^{+}\left[V\left(a,f\right)+{\bar{N}}_{k}\left(r,a\right)\right]}{\log r};\\ \bar{\rho }\left(a,f\right)=\underset{r\to \infty }{\mathrm{limsup}}\frac{\log^{+}\left[V\left(a,f\right)+\bar{N}\left(r,a\right)\right]}{\log r};\\ \rho \left(a,f\right)=\underset{r\to \infty }{\mathrm{limsup}}\frac{\log^{+}\left[V\left(a,f\right)+N\left(r,a\right)\right]}{\log r}. \end{array}$$

We say that *a* is an

- *E*-valued evB (exceptional value in the sense of Borel) for *f* for distinct zeros of order ≤*k* if $\;{\bar{\rho }}_{k}(a,f)<\lambda (f)$;
- *E*-valued evB for *f* for distinct zeros if $\;\bar{\rho }(a,f)<\lambda (f)$;
- *E*-valued evB for *f* (for the whole aggregate of zeros) if *ρ*(*a*, *f*) < *λ*(*f*).

Suppose that *f*(*z*) is an *E*-valued meromorphic function with finite order *ρ* > 0 in *ℂ*. Xuan and Wu [7] proved that the order of *f*′ is *ρ*. Hence for any positive integer *l* the order of *f* ^(*l*)^ is *ρ*. Therefore, we call *a* a vector-valued evB for *f* ^(*l*)^ for distinct zeros of order ≤*k*, if

(34) ρ¯k(a,f(l))=limsup⁡r→∞log⁡[V(r,a,f(l))+N¯k(r,a,f(l))]log⁡r<ρ.

In this section, we will prove the following theorem.

**Theorem 6**

Let *f*(*z*) be an admissible *E*-valued meromorphic function of compact projection in *ℂ* and the order of *f*(*z*) is *ρ* (0 < *ρ* < +*∞*). Suppose that $\hat{\infty }$ is an *E*-valued evB for *f* for distinct zeros of order ≤*l*, *a* ^[*i*]^ ∈ *E* (*i* = 1,2,…, *p*) are *E*-valued evB for *f* for distinct zeros of order ≤*m*_*i*_, and *b* ^[*j*]^(≠0) ∈ *E* (*j* = 1,2,…, *q*) are *E*-valued evB for *f* ^(*k*)^ for distinct zeros of order ≤*n*_*j*_, where *k*, *p*, *q*, *l* and all of *m*_*i*_, *n*_*j*_ are positive integers. Then

(35) $$\begin{array}{c} \overset{p}{\underset{i=1}{\sum }}\frac{kq+1}{m_{i}+1}+\overset{q}{\underset{j=1}{\sum }}\frac{1}{n_{j}+1}+\frac{1}{l+1}\left(1+k\overset{q}{\underset{j=1}{\sum }}\frac{1}{n_{j}+1}\right)\geq pq. \end{array}$$

**Proof**

By Theorem 3, we obtain

(36) {pq−(∑i=1pkq+1mi+1     +∑j=1q1nj+1+1l+1(1+k∑j=1q1nj+1))}T(r,f)≤ll+1(1+k∑j=1q1nj+1)N¯l(r,f) +(kq+1)∑i=1p{N¯mi(r,a[i],f)+V(r,a[i],f)} +∑j=1q{N¯nj(r,b[j],f(k))+V(r,b[j],f(k))}+S(r,f).

Since $\hat{\infty }$ is an *E*-valued evB for *f* for distinct zeros of order ≤*l*, *a* ^[*i*]^ ∈ *E*  (*i* = 1,2,…, *p*) is an *E*-valued evB for *f* for distinct zeros of order ≤*m*_*i*_ and *b* ^[*j*]^(≠0) ∈ *E*  (*j* = 1,2,…, *q*) is an *E*-valued evB for *f* ^(*k*)^ for distinct zeros of order ≤*n*_*j*_. Thus there is a 0 < *μ* < *ρ* such that for any *i*, *j*  (*i* = 1,2,…, *p*; *j* = 1,2,…, *q*) we have

(37) $$\begin{array}{c} {\bar{N}}_{l}\left(r,f\right)\leq R^{\mu },\\ {\bar{N}}_{m_{i}}\left(r,a^{\left[i\right]},f\right)+V\left(r,a^{\left[i\right]},f\right)\leq R^{\mu },\\ {\bar{N}}_{n_{j}}\left(r,b^{\left[j\right]},f^{\left(k\right)}\right)+V\left(r,b^{\left[j\right]},f^{\left(k\right)}\right)\leq R^{\mu }. \end{array}$$

It follows from 0 < *μ* < *ρ* and (36) and (37) that

(38) $$\begin{array}{c} \overset{p}{\underset{i=1}{\sum }}\frac{kq+1}{m_{i}+1}+\overset{q}{\underset{j=1}{\sum }}\frac{1}{n_{j}+1}+\frac{1}{l+1}\left(1+k\overset{q}{\underset{j=1}{\sum }}\frac{1}{n_{j}+1}\right)\geq pq. \end{array}$$

Letting *p* = *q* = 1 in Theorem 6, we can get the following corollary.

**Corollary 7**

Let *f*(*z*) be an admissible *E*-valued meromorphic function of compact projection in *ℂ* and the order of *f*(*z*) is *ρ* (0 < *ρ* < +*∞*). Suppose that $\hat{\infty }$ is an *E*-valued evB for *f* for distinct zeros of order ≤*l*, where *l* is an integer ≥1. If there exist *a*, *b* ∈ *E*, *b* ≠ 0, such that *a* is an *E*-valued evB for *f* for distinct zeros of order ≤*p* and *b* is a an *E*-valued evB for *f* ^(*k*)^ for distinct zeros of order ≤*q*, where *p*, *q* are positive integers, then

(39) $$\begin{array}{c} \frac{q+1+k}{\left(q+1\right)\left(l+1\right)}+\frac{k+1}{p+1}+\frac{1}{q+1}\geq 1. \end{array}$$

If $\hat{\infty }$, *a* are *E*-valued evB for *f* for distinct zeros, that is, letting *l*, *p* tend to infinity in (39), we can get 1/(*q* + 1) ≥ 1. This means that, for each integer *k*, *q* ≥ 1,$\;\;{\bar{\rho }}_{q}(b,f^{\left(k\right)})\geq \rho$, for all $b\neq 0,\neq \hat{\infty }$. Hence, we can get the following corollary.

**Corollary 8**

Let *f*(*z*) be an admissible *E*-valued meromorphic function of compact projection in *ℂ* and the order of *f*(*z*) is *ρ* (0 < *ρ* < +*∞*). Suppose that $\hat{\infty }$, *a* ∈ *E* are *E*-valued evB for *f* for distinct zeros. Then, for all positive integers *k* and *q*, ${\bar{\rho }}_{q}(b,f^{\left(k\right)})=\rho$ for all $b\neq 0,\neq \hat{\infty }$.

The corresponding results of Corollaries 7 and 8 for the meromorphic scalar value function were obtained by Gopalakrishna and Bhoosnurmath [14] and Singh and Gopalakrishna [15]. The corresponding results of Corollaries 7 and 8 for the meromorphic scalar value function on annuli were obtained by Chen and Wu [16].
